# *Tectus niloticus* (Tegulidae, Gastropod) as a Novel Vector of Ciguatera Poisoning: Clinical Characterization and Follow-Up of a Mass Poisoning Event in Nuku Hiva Island (French Polynesia)

**DOI:** 10.3390/toxins10030102

**Published:** 2018-02-28

**Authors:** Clémence Mahana iti Gatti, Davide Lonati, Hélène Taiana Darius, Arturo Zancan, Mélanie Roué, Azzurra Schicchi, Carlo Alessandro Locatelli, Mireille Chinain

**Affiliations:** 1 Laboratory of Toxic Microalgae, Institut Louis Malardé (ILM)—UMR 241-EIO, P.O. box 30, 98713 Papeete, Tahiti, French Polynesia; tdarius@ilm.pf (H.T.D.); mchinain@ilm.pf (M.C.); 2 Poison Control Centre and National Toxicology Information Centre—Toxicology Unit, Istituti Clinici Scientifici Maugeri, IRCCS Maugeri Hospital and University of Pavia, 27100 Pavia, Italy; davide.lonati@icsmaugeri.it (D.L.); azzurra.schicchi@icsmaugeri.it (A.S.); carlo.locatelli@icsmaugeri.it (C.A.L.); 3 Subacute Care Unit, Istituti Clinici Scientifici Maugeri, IRCCS Maugeri Hospital, 27100 Pavia, Italy; arturo.zancan@icsmaugeri.it; 4 Institut de Recherche pour le Développement (IRD)—UMR 241-EIO, P.O. box 529, 98713 Papeete, Tahiti, French Polynesia; melanie.roue@ird.fr

**Keywords:** ciguatera poisoning, *Tectus niloticus*, ciguatoxins, health hazards, clinical follow-up, neurological exploration, French Polynesia

## Abstract

Ciguatera fish poisoning (CFP) is the most prevalent non-bacterial food-borne form of poisoning in French Polynesia, which results from the consumption of coral reef fish naturally contaminated with ciguatoxins produced by dinoflagellates in the genus *Gambierdiscus*. Since the early 2000s, this French territory has also witnessed the emergence of atypical forms of ciguatera, known as ciguatera shellfish poisoning (CSP), associated with the consumption of marine invertebrates. In June 2014, nine tourists simultaneously developed a major and persistent poisoning syndrome following the consumption of the gastropod *Tectus niloticus* collected in Anaho, a secluded bay of Nuku Hiva Island (Marquesas Archipelago, French Polynesia). The unusual nature and severity of this event prompted a multidisciplinary investigation in order to characterize the etiology and document the short/long-term health consequences of this mass-poisoning event. This paper presents the results of clinical investigations based on hospital medical records, medical follow-up conducted six and 20 months post-poisoning, including a case description. This study is the first to describe the medical signature of *T. niloticus* poisoning in French Polynesia and contributed to alerting local authorities about the potential health hazards associated with the consumption of this gastropod, which is highly prized by local communities in Pacific island countries and territories.

## 1. Introduction

Marine products represent a major subsistence resource for many Pacific Island countries and territories (PICTs) where the risk of seafood poisoning is likely to pose significant health and economic issues to local populations [1]. Ciguatera fish poisoning (CFP) is related to the consumption of coral reef fish from tropical and subtropical regions that are contaminated by ciguatoxins (CTXs), a family of neurotoxins produced by discoid-shaped benthic dinoflagellates of the genus *Gambierdiscus* [2].

To date, CFP is the most prevalent ichthyosarcotoxism reported in French Polynesia [3,4] with an annual average incidence rate of 19/10,000 inhabitants, although this number may reach 1700/10,000 inhabitants in the most severely affected islands. The annual prevalence of CFP worldwide has been estimated to be around 50,000–100,000 cases, but these statistics could represent only 20% of actual cases, due to misdiagnosis and under-reporting [2]. CFP is endemic in the Caribbean [5], Indo-Pacific Islands [1,4], and Indian Ocean [6]. However, recent indigenous CFP cases have been reported in Spain (Canary Islands) [7], temperate areas of Japan, South Korea [8,9], and CTX-containing marine organisms have been reported in the Madeira Archipelago and Israeli coast [10,11], reflecting a progressive expansion of the ciguatoxic risk at a global scale. Moreover, due to the increase in the international fish trade, tourism, and interest in exotic products, non-endemic regions, such as Europe or North America, are currently facing an increase in imported CFP cases [12,13,14], raising new concerns about fish import regulations, medical professional outreach, and management of a new category of patients.

CTXs are highly lipophilic polyethers that bind primarily to the voltage-gated sodium channels (VGSCs) and are also implicated in voltage-gated potassium channels (VGKCs), calcium channels (VGCCs), and the inducible nitric oxide synthase (iNOS) disruptions responsible for significant excitable cell disturbances at very low concentrations (picomolar to nanomolar) [15,16,17,18,19,20,21]. Due to the wide distribution of CTXs’ biological targets and the large variety of tissues affected, CFP is characterized by highly polymorphic, non-specific clinical signs with more than 175 different symptoms reported so far [22], which makes diagnosis difficult to establish, especially in non-endemic regions.

CFP expresses through a combination of digestive, cardiovascular, neurological, and general clinical manifestations [23]. Common gastrointestinal effects include diarrhea, vomiting, and abdominal pain. These symptoms generally appear within 12 h and resolve after 24–48 h [4]. Cardiovascular disturbances (hypotension, bradycardia), are less prevalent, and are suggestive of the severity of the poisoning [24,25]. Other initial symptoms may include a general malaise, pain and weakness of the lower limbs, intense pruritus without cutaneous signs, arthralgia and paresthesia (typically perioral and in the extremities), in the absence of fever [25,26]. Among neurologic symptoms, cold allodynia (dysesthesia in response to cold stimulations), commonly reported as “temperature reversal”, is a specificity considered nearly pathognomonic of CFP [27,28,29]. Although fatal cases of CFP remain anecdotal (<0.1%) [30], this disease may prove extremely debilitating and deeply distressing in the short- or long-term (months or years) [31,32,33].

According to a few studies, the prevalence of CFP patients who develop chronic manifestations is up to 20% [34,35]. However, the lack of a clear definition of “chronic” CFP, difficulties in correlating long-term manifestations with previous CTX exposure, and the absence of specific biomarkers or diagnostic tools, make it extremely difficult to obtain a reliable estimation of the prevalence of this phenomenon. In the absence of a general consensus, chronic CFP is usually defined as the persistence of symptoms for more than 2 to 6 months. Additionally, clinical manifestations are not specific and can easily mimic chronic fatigue syndrome, as well as chronic systemic and inflammatory illnesses [36]. The manifestations of chronic CFP are mainly neurological (paresthesia, dysesthesia, cold allodynia, itching, headache), psychiatric (cognitive dysfunction, sleep disorder, anxiety, memory loss, depression), and systemic (myalgia, severe asthenia, arthralgia) [23,25,26,28,33,37]. These manifestations can be expressed continuously, more or less intensely, or by “crises” triggered by the consumption of certain food (marine products, meat, nuts), drink (alcohol, coffee), and even under specific behavioral and environmental circumstances, such as intense physical activity or stress, and ambient temperature or pressure variations [23,38,39].

Additionally, since the early 2000s, French Polynesia has also witnessed the emergence of atypical forms of CFP, designated as ciguatera shellfish poisoning (CSP) and associated with the consumption of marine invertebrates, such as giant clams and sea urchins, which are very popular among local populations. So far, the formal implication of CTXs in this phenomenon are still unclear as recent studies conducted in New Caledonia, Vanuatu, and French Polynesia identified cyanobacteria as the potential toxin-source organisms of CSP [40,41], although several studies confirmed the ability of giant clams (*Tridacna maxima*), starfish (*Ophidiaster ophidianus*, *Marthasterias glacialis)*, sea urchins (*Tripneustes gratilla*), lobsters **(***Panulirus penicillatus*) and octopus (*Octopodidae*) to bioaccumulate CTXs [42,43,44,45]. These observations suggest that CSP could actually result from the exposure of consumers to multiple marine toxins, depending on the invertebrate’s metabolization abilities. Indeed, clinical manifestations of CSP include symptoms typical of CFP, e.g., gastrointestinal (diarrhea, vomiting), neurological (paresthesia, dysesthesia), and cardiovascular (bradycardia, hypotension) symptoms. However, distinguishable clinical signs are also observed, such as early onset and severity of symptoms, occasionally followed by transient paralysis [41]. Until 2014, marine invertebrates implicated in ciguatoxic syndrome reported in French Polynesia mostly involved giant clams and sea urchins [43].

**Figure 1:**
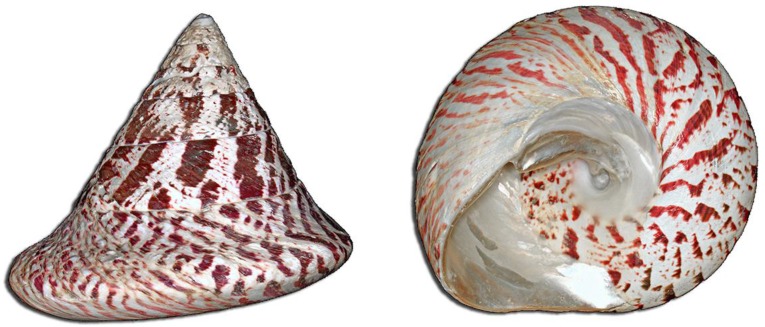
*Tectus niloticus* (Linnaeus, 1767) (photo credit: © H. Zell).

*Tectus niloticus* (Linnaeus, 1767) (Figure 1), also known as trochus, is a marine gastropod grazer from the *Tegulidae* family [46] introduced in French Polynesia in 1957 for its commercial exploitation (food, craft, cosmetic) [47,48]. *T. niloticus* is now a protected species and is subject to regulated fishing campaigns organized by local authorities for trade and personal consumption [49]. Although anecdotal or unofficial trochus poisonings are mentioned by local populations in the South Pacific [50], no CSP cluster directly associated to the consumption of this gastropod has been formally reported so far in French Polynesia, and to date, no clinical descriptions are available.

In June 2014, eleven marine tourists shared a meal of trochus collected in Anaho Bay (Nuku Hiva Island, Marquesas Archipelago). Nine of them developed severe manifestations evocative of a CFP. The unusual nature of this mass-poisoning event, characterized by involvement of an atypical marine organism and severe clinical manifestations, prompted a comprehensive study of this poisoning event. This utilized a multidisciplinary approach including: (i) medical investigations and follow-up of the nine patients, (ii) field investigations aiming at monitoring the toxicity of *T. niloticus* specimens collected from Anaho Bay, and (iii) characterization of the toxins involved in this event [51].

The present work illustrates a bed to bench translational research approach. It provides a detailed description of the short- and long-term clinical signature of this poisoning in patients, and highlights the difficulty in managing the disease, both in endemic and non-endemic regions.

## 2. Results

### 2.1. Patients Description and Acute Clinical Manifestations

As shown in Table 1, the poisoning outbreak affected three women and six men, from 39 to 72 years-old (y/o), in “excellent” health (i.e., no apparent health problems), except for two of them who were declared to be in “good” health (i.e., with minor or limited health problems). Prior to the poisoning outbreak, patient no. 2 suffered from renal colic, while patients no. 3 and no. 6 suffered from chronic hypertension.

First manifestations of the poisoning appeared within 2 to 4 h, including profuse diarrhea, vomiting, and abdominal pain with hiccups for two patients and esophagitis for patient no. 9. Except for patients no. 2 and no. 9 who experienced digestive disorders for more than 1 month, all other patients felt relief after 48 h. Seven out of nine patients presented with moderate to severe hypotension accompanied with bradycardia for six of them, and three showed heart rhythm disorder. All cardiovascular manifestations disappeared within 72 h. Except for patient no. 5, who did not show neurological manifestations during the acute phase of the poisoning, all other patients presented with intense cold allodynia, tingling of extremities and burning of the throat/mouth. Itching, touch disturbances, and dysgeusia were also predominant. Other neurological disturbances included dizziness, headache, vision, language and balance disorders, and anxiety.

Among general manifestations, severe asthenia, myalgia, urogenital disturbances (burning, itching, pain, discomfort), arthralgia and feet swelling, and burning or pain, were experienced by a majority of patients. Although less frequently observed, hypothermia, shivers, and sleep disorder were also part of the acute manifestations of the poisoning.

Six weeks after the poisoning, following a gradual loss of muscle strength and a fainting episode, patient no. 8 was diagnosed with a polyradiculoneuritis, which prompted his hospitalization at the Hospital Center of French Polynesia (Tahiti).

### 2.2. Six- and 20-Month Medical Follow-Up

#### 2.2.1. Chronic Symptoms

As shown in Table 2, six months after the poisoning event, all patients declared no chronic cardiovascular disorders and only patient no. 2 showed occasional diarrhea. In contrast, all patients confirmed experiencing recurrent neurological disorders, such as tingling of the extremities, touch disturbance, cold allodynia, or itching, and, less frequently, burning of the throat/mouth, urogenital disturbances, dysgeusia, and balance disorder. The majority of patients also declared that they struggled with chronic asthenia and sleep disorder. Myalgia, shivers, irritability, hypothermia, and feet swelling/burning/pain, were only experienced by a limited number of patients.

Twenty months post-poisoning, chronic manifestations eventually resolved in six out of nine patients and significantly decreased in intensity in patients no. 4, 7, and 8, who still sporadically experienced moderate to light tingling of the extremities, cold allodynia, itching, burning of the throat and mouth, sleep disorder, feet swelling/burning/pain, or restless legs syndrome. Only patient no. 8 still presented with permanent touch disturbance after 20 months and has developed a carpal tunnel syndrome on both sides (data not shown). Finally, patients no. 7 and no. 8 both developed a vulnerability to compression neuropathy (data not shown).

#### 2.2.2. Recurrence of Symptoms and Triggering Factors

As shown in Table 3, all patients identified triggering factors responsible for transient recurrence or intensification of clinical manifestations 6 months post-poisoning, which consisted mainly of tingling of extremities, itching and burning sensations. Among food-based factors, alcohol and beef meat consumption were mostly cited and, to a lesser extent, fish, pork, nuts, foods rich in protein, cheese and salted biscuits. They also identified situations or behaviors such as physical activity, severe variation of ambient temperature, fatigue, lack of sleep, rapid weight loss, wind and sun exposure as other factors responsible for manifestations’ resurgence or intensification.

Over time, patients’ “hypersensitivity” to these factors significantly decreased or eventually disappeared. However, 20 months after their poisoning, six out of nine patients still reacted to alcohol, two to fatigue/lack of sleep and only one to beef meat (data not shown).

#### 2.2.3. Medical Management of Acute and Chronic Symptoms in Patients No. 1–8

Following their admission to Louis Rollin Hospital in Nuku Hiva, patients no. 1–6 received parenteral hydration, cetirizine (a class H1 antihistaminic) and paracetamol that contributed to significantly diminish cardiovascular and digestive manifestations. According to the patients, administration of dexchlorpheniramine maleate (a first generation antihistaminic) resulted in significant improvement of itching in patients no. 3, 4 and 6, but was unsuccessful for patients no. 1, 2, 7 and 8. Several weeks after the poisoning, patient no. 1 drank a traditional remedy based on a decoction of *Heliotropium fortherianum* leaves (commonly used by South Pacific populations to treat ciguatera [52]) but without any improvement. The polyradiculoneuritis of patient no. 8, diagnosed six weeks after the poisoning was treated with gammaglobulin injections which significantly improved his health condition. As reported by patients no. 7 and no. 8, gabapentin (an anticonvulsant and antiepileptic) and amitriptyline (a tricyclic antidepressant) were inefficient in treating their chronic neurological symptoms, unlike the zolpidem (a sedative-hypnotic) that significantly improved sleep disorder. Paracetamol helped to improve chronic itching crises according to patients no. 1 and no. 2.

In addition to treatments provided at the Louis Rollin Hospital, Nuku Hiva, patients did receive diet recommendations post-hospitalization. This diet included complete removal of all marine products for one month and a reduction in eggs, red meat, chicken, pork meat, canned products and alcohol consumption for ten days.

Finally, 20 months after the poisoning, all patients declared they no longer took any medication in relation to the manifestations of the initial poisoning.

### 2.3. Index Case Description (Patient No. 9)

Patient no. 9, a man of 45 y/o without a history of comorbidities, developed severe vomiting, diarrhea, abdominal pain, bradycardia, and hypotension, 2 h after the ingestion of the toxic meal. As a first-line treatment, at the closest infirmary to Anaho Bay, he received rehydration therapy, paracetamol, and ephedrine, pending his transfer to Louis Rollin Hospital of Nuku Hiva, where he was diagnosed as suffering from ciguatera poisoning (encoded T61.0 according to the International Statistical Classification of Diseases and Related Health Problems 10th Revision, ICD-10). At his admission to Nuku Hiva hospital, extensive biological investigations revealed a hypokalemia, a hyponatremia, and a neutrophil polynucleosis. Hepatic biological parameters were normal. He was treated with the administration of fluids, metoclopramide, esomeprazole, hydroxyzine, and phloroglucinol/trimethylphloroglucinol. While this significantly improved the gastrointestinal symptoms and vomiting, the patient kept experiencing invalidating dysgeusia, dysphagia, and incoercible hiccups that led to his transfer to the Hospital Center of French Polynesia (Tahiti), where he underwent an esophagogastroduodenoscopy that revealed a grade 2 esophagitis. Bacterial investigations turned out negative. After four days of hospitalization in Tahiti, the patient went back to Italy, his home country, and was admitted to a peripheral hospital for two weeks. Due to the persistence of digestive and neurological manifestations, the patient was transferred to the Pavia Poison Control Centre, National Toxicology Information Centre (PPCC) for extensive investigations and specialist surveillance. Considering the origin of the poisoning, the nature, and severity of symptoms, in addition to the continuation of symptomatic treatment (fluids and proton-pump inhibitors), high doses of mannitol (1 g/kg/day, intravenously) were administered for a week. A progressive resolution of digestive disorders was observed after 48 h, but neurological manifestations such as shivers, hypothermia, and severe dysesthesia with paradoxical heat perception worsened/appeared. An electroneuronograph performed six weeks after the poisoning, revealed an axonal polyneuropathy in the lower limbs. A thermal-quantitative sensory test (QST) confirmed hypoesthesia for warm stimuli and cold allodynia. An electromyography, tested for latent tetany and prolonged ischemia, remained normal, and epidermal skin-biopsy showed normal nerve fiber density. Considering the neurological disorders persistence and the lack of responsiveness to mannitol, carbamazepine (400 mg/day) was started at week seven, once HLA-A*3101 hyper-sensibility was excluded (in order to avoid a hypersensitivity reaction, Stevens–Johnson syndrome, toxic epidermal necrolysis, or death of the patient) [53]. Three months post-poisoning, a persistent class A esophagitis (Los Angeles classification) [54] was still present. Asthenia, myalgia, hypothermia, shivers, paresthesia, cold allodynia, urogenital disturbances, irritability, and sleep disorders were still persistent six months after the poisoning. Finally, over time all manifestations related to the poisoning spontaneously decreased in intensity and eventually resolved after twenty months.

## 3. Discussion

The present study is the first to describe a mass-poisoning event related to the consumption of the gastropod *Tectus niloticus*, which occurred in Anaho Bay, Nuku Hiva Island (Marquesas Archipelago, French Polynesia), a site known to harbor highly-toxic fish specimens (data not shown) with CFP incidence rates varying from 37 to 101/10,000 inhabitants between 2012 and 2016.

In the present study, the acute symptoms reported in the nine patients strongly evoked CFP with a first-line combination of transient digestive (diarrhea, vomiting) and cardiovascular (bradycardia, hypotension) symptoms, which quickly resolved in most patients. These were followed by secondary neurological/general manifestations, such as itching, dysesthesia, paresthesia, asthenia, myalgia, hypersensitivity to certain food and environmental factors, and cold allodynia (intense and painful tingling, burning, or electric sensation in response to a cold stimulus [29]), which can be considered as pathognomonic of this poisoning [23,27]. The high polymorphism of symptoms observed, results from both the wide distribution of CTXs’ biological targets, i.e., VGSCs, VGKCs, VGCCs, as well as CTXs’ indirect effects on Na^+^- Ca^2+^ exchange, and the diversity of excitable cells affected by CTXs (peripheral nervous system, sensory neurons, skeletal muscle, heart, brain) [15,18,19,20,29,55,56]. Seven out of nine patients suffered from urogenital disturbances, such as itching, burning sensation, or pain. It has been suggested that these peculiar manifestations could be associated with the excretion mechanism of CTXs in the same way they are excreted in urine and feces [57]. This phenomenon could explain the transmission of genital pruritus often observed after sexual intercourse between a CFP patient and his/her partner, leading some authors to suggest that CFP could be a “sexual transmitted disease” [58].

Among central neurological manifestations, balance, language, and vision disorders, hypothermia, irritability, and anxiety are reported following the acute phase. These could be due to the direct action of CTXs on the central nervous system, since it has been shown in an animal model that CTX administration can lead to brain cortex neuronal excitotoxicity [21] and be responsible for cerebellar syndrome in CFP patients [3].

Hypothermia episodes described in CFP patients and in the study population may correspond to the biological response of the thermoregulation system to CTX exposure [59]. Finally, the esophagitis developed by patient no. 9 immediately after the poisoning, and which was still present six months after the toxic event, could result from the intense vomiting episodes and be responsible for their incoercible hiccup.

Six weeks after the poisoning, patient no. 8 was diagnosed with a polyradiculoneuritis, an autoimmune disease that targets the peripheral nervous system. Even if no direct evidence has been established between CFP and such peripheral neuropathy, cases of Guillain-Barré syndrome and polyradiculoneuritis have already been reported consecutively to CFP [3,32]. It has been suggested that the swelling of the nerve cells caused by CTX activity, leading to a degradation of the myelin sheath, could be the cause of a form of autoimmunity directed against the peripheral nervous system and be responsible for disturbances evocative of a polyradiculoneuritis [32,60]. Moreover, the axonal polyneuropathy of lower limbs detected in patient no. 9 and the compression neuropathy developed by patients no. 7 and no. 8, illustrate a long-term consequence of nerve cell degradation and sensitization caused by exposure to CTXs.

Although it is still poorly documented, chronic manifestations of CFP are reported in at least 20% of patients [28,34,35]. In the present study, all patients experienced recurrent/persistent manifestations six months after the poisoning, and for three of them (30%), over a period of twenty months. Chronic manifestations of CFP are primarily neurological or general, and are expressed through a combination of paresthesia, dysesthesia, pruritus, cold allodynia, asthenia, general malaise, and depression [23]. As observed in the present study, chronic disturbances often spontaneously decrease and eventually resolve, but the long-term duration, which can extend to several years according to the literature [37], may vary from one individual to another and cannot be predicted based on current knowledge. This phenomenon could result from the prolonged activation of voltage-gated sodium channels by CTXs, associated, or not, to an immune dysregulation. Indeed, two hypotheses are currently debated: (i) the first is based on the ability of CTXs to be stored in deep tissue and occasionally released into the blood stream following lipid metabolism activation [61], which explains the resurgence of symptoms observed after rapid weight loss in patients no. 1, 2, 5, and 6; and (ii) the second relies on an immunological imbalance common to chronic auto-inflammatory and autoimmune diseases. Indeed, recent studies conducted in chronic CFP patients suggested that the HLA genetic profile may play a significant role in this pathogenesis, and HLA typing may be used as a potential predictor of ciguatera poisoning chronicity. Moreover, the authors identified HLA patterns associated with the immune system imbalance in chronic CFP patients that are responsible for long-term inflammatory conditions [62,63,64,65,66].

Another particularity of CFP, also observed in the study population, concerns the modulation of expression of both acute and chronic clinical manifestations by specific triggering factors responsible for rapid and transient recurrence or intensification of neurological disturbances. Over-reaction to food, a beverage, or situation is often reported in the literature [23,33], but the mechanisms underlying this “hypersensitivity” are still unclear. In CFP-endemic countries, a CFP patient’s medical management is always associated with a specific diet aiming at limiting further consumption of marine products, animal proteins, alcohol, nuts, and food rich in fat or histamine, for at least one month, or as long as the patient experiences adverse reactions to food products [23,67]. As observed in the present study, this hypersensitivity spontaneously decreased and eventually resolved over time.

To date, no specific drug or medical protocol has proved to be clearly efficient in treating acute or chronic manifestations of CFP. Medical management essentially relies on supportive care, including antispasmodic, antiemetic, anti-diarrheic, cardioactive drugs (atropine), chronotropic pressors (epinephrine), fluids, calcium gluconate injection, activated charcoal, paracetamol, nifedipine or vitamin complex administration (B1, B6, B12), [23,39,68,69]. Pruritus expressed in CFP does not seem to be mediated by the histamine pathway, but could rather result from sensory nerve fiber dysfunction [27], which explains the lack of efficiency of certain anti-histaminic drugs. Nevertheless, H1-class anti-histaminic drugs display better results on pruritus, probably because of an indirect consequence of their sedative effects. Hospitalization is recommended in cases of severe hydro-electrolytic disturbances and for at-risk populations, such as the elderly, pregnant women, or patients suffering from chronic cardiovascular pathology prior to the poisoning [23].

For years, intravenous administration of mannitol was recommended as a first-line treatment of neurological symptoms in acute CFP [70,71]. Indeed, descriptive reports and a few randomized blinded clinical trials showed mannitol administration resulted in significant improvement in CFP patients’ health conditions [37,71,72], provided it is taken within 48–72 h after the poisoning for optimal results [70,72]. It has been observed that mannitol helps to reduce nerve cell swelling following CTX exposure, and also suggested that it acts as a scavenger of free radicals generated by CTXs [73]. Although the clinical efficiency mannitol remains controversial [74], it seems reasonable to consider its use in the treatment of the acute phase of the poisoning [75]. In the present study, patient no. 9 received mannitol treatment several weeks after the poisoning, which may explain the lack of improvement.

Antidepressant drugs (amitriptyline, fluoxetine) [69,76], antiepileptics (gabapentin, pregabalin) [77,78], and non-steroidal anti-inflammatory agents and cholestyramine (basic anion-exchange resin) [62] have tentatively been proposed to treat CFP chronic manifestations. These small case series studies do suggest efficiency on chronic symptoms, but some of yielded inconsistent results and needed several weeks’ administration of these drugs, which often leads to deleterious side effects. Moreover, symptoms may reappear after these treatments are stopped [78]. The lack of comparative trials and clear evidence of their efficiency makes it difficult to provide clear recommendations. In the present case, neither gabapentin nor amitriptyline showed efficiency in treating chronic manifestations in patients no. 7 and no. 8. In any case, the persistence of symptoms for months and the lack of clear improvement following patient no. 9’s several hospitalizations highlight the complexity of medical management of this neglected tropical pathology, both in endemic and non-endemic regions.

Several weeks after the poisoning, one of the patients (no. 1) eventually turned to a local remedy based on a decoction of *Heliotropium foertherianum* leaves, but without beneficial effects. This preparation is traditionally used by South Pacific communities to treat CFP, especially those living in remote islands with limited access to medical facilities [52]. In vitro studies suggest that this traditional preparation has the ability to compete with CTXs, thus limiting their fixation on their biological targets [79], which may explain why this remedy is usually recognized to be effective only when taken promptly after the first signs of the poisoning, and the lack of symptoms’ improvement in patient no. 1.

In non-endemic regions, diagnosis can take weeks and various expert consultations, which can be a source of anxiety for the patient who does not know what he/she is suffering from, or how the symptoms will progress. Moreover, the lack of recognition of this disease by medical professionals, due to the high subjectivity of manifestations, not perceptible to others, is a real struggle for some patients. It has been suggested to clinically define ciguatera poisoning as a combination of digestive, cardiovascular, and neurological symptoms, occurring within 48 h after consumption of reef organisms, in the absence of fever, rash, or any allergic manifestations. In addition, although cold allodynia is presented as a pathognomonic manifestation of CFP [28], it is sometimes reported in the poisoning syndrome related to brevetoxin exposure, a toxic compound structurally comparable to CTXs [80]. In this case, the origin of the poisoning, i.e., mollusk vs. fish, can help discriminate both poisonings, but implies a good knowledge of marine toxin-related syndromes. Hence, in the context of globalization of CFP/CSP, it seems essential to improve the outreach of medical professionals, especially in non-endemic regions, and to develop objective diagnosis tools.

In the present study, toxicological investigations, using both functional and chromatographic analysis, were also conducted on *T. niloticus* specimens collected from Anaho Bay, one, six, and 28 months after this poisoning event and confirmed the presence of CTX compounds [51]. The doses observed in trochus specimens tested individually exceeded 10 ng equiv. CTX/g of flesh, which explains the severity of the symptoms among this study population. Although CTX content in trochus specimens progressively decreased over this survey period, it remained above the safety limit recommended for human consumption, even 28 months after the toxic event [51]. Lastly, the burning of the mouth/throat and dysgeusia experienced by eight out of nine patients is rarely reported in classical CFP within the literature [23] and could testify to the presence of other toxic compounds, which also explains the particularly rapid onset of symptoms (between 2 and 4 h). Concomitant exposure to multiple toxins should be considered and further investigations are ongoing in order to explore this possibility. Moreover, it was recommended that systematic monitoring of trochus toxicity be conducted prior to the collection campaigns authorized annually by the Marine and Mineral Resources Directorate of French Polynesia, in order to avoid/limit new epidemics.

Finally, French Polynesia is among the very few countries in the Pacific to have implemented an epidemiological surveillance program to collect information about human ciguatera poisoning cases. However, reporting of CFP is still not mandatory and there is currently no regulation imposed on fishermen or suppliers to check for the presence of marine biotoxins in seafood prior to sale. However, the Institut Louis Malardé (Tahiti, Society Archipelago, French Polynesia) does get occasional requests from public services, supermarkets, fishermen or private individuals, to perform toxicological analyses in the following circumstances: (i) check for the presence of CTXs in food remnants involved in poisoning incidents/outbreaks, (ii) check for the CFP risk status of lagoon areas which have undergone extensive construction/development works, (iii) check for the absence of CTXs in seafood on sale. Discussions are ongoing with stakeholders from the Public Health Directorate of French Polynesia to render CFP reporting mandatory and to impose regular monitoring of the toxic status of main fishing zones or recurrent toxic species sold in public markets and supermarkets. Unfortunately, these precautions will not prevent poisoning associated with family/artisanal fishing, which is preponderant in French Polynesia.

## 4. Conclusions

This study is the first to describe the medical signature of a poisoning outbreak following the ingestion of *Tectus niloticus* contaminated with CTXs, thus confirming this gastropod as a novel vector of CFP in French Polynesia. This event was characterized by severe and prolonged gastrointestinal, cardiovascular, and neurological symptoms, as well as the persistence of clinical manifestations over a long period of time, influenced by both food- and behavioral-based triggering factors. Also, this event further prompted local authorities to display signs banning fishing activities and the consumption of marine products from Anaho Bay. Alert messages were also broadcasted as necessary on local televisions, radios and in newspapers. This poisoning incident helped to greatly improve public awareness about this emerging toxic threat among local populations and public health authorities. In conclusion, these findings raise the importance of (i) implementing a region-wide surveillance program on the toxicity of *T. niloticus* and, more generally, of marine products which often represent both a significant subsistence resource and a source of income in PICTs; (ii) improving public and medical professionals’ awareness (particularly in non-endemic regions) in order to minimize the risk of poisoning outbreaks, improve diagnosis, and medical management of patients; and (iii) stimulating additional studies aiming at identifying suitable and effective treatments.

## 5. Materials and Methods

### 5.1. Context

**Figure 2:**
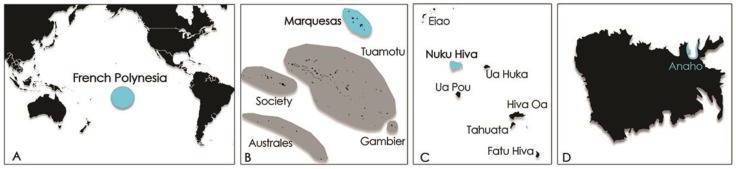
Map of (**A**) French Polynesia; (**B**) Marquesas archipelago; (**C**) Nuku Hiva Island; and (**D**) Anaho Bay.

On 19 June 2014, eleven marine tourists shared a meal of a dozen of *Tectus niloticus* (trochus), collected in Anaho Bay, Nuku Hiva Island, Marquesas Archipelago (Figure 2).

Nine of the patients (two French, two Dutch, five Italian) developed a severe poisoning syndrome that led to the admission of six of them (patients no. 1, 2, 3, 4, 6, and 9) to the Louis Rollin Hospital in Nuku Hiva, the day after the poisoning event, with severe gastrointestinal, cardiovascular, and neurological manifestations, while patients no. 7 and no. 8, both physicians, preferred to treat themselves. At admission, patients were diagnosed with ciguatera fish poisoning (CFP) (encoded T61.0 according to the International Statistical Classification of Diseases and Related Health Problems, 10th Revision (ICD-10)), and a specific reporting form was filled in for each patient. This form was jointly developed by the Institut Louis Malardé (Papeete, Tahiti) and the Public Health Directorate of French Polynesia, within the framework of the CFP Epidemiological Surveillance Network on-going in French Polynesia since 2007. As part of this surveillance program, all public health medical professionals are invited to report each CFP case diagnosed or suspected in a patient. This standardized clinical form provides information about the date of marine product collection and consumption, the species implicated, parts consumed, name of the supplier of the marine products or fishing location, incubation time, cardiovascular constants, symptoms developed during the acute phase of the poisoning, and any supplementary information that the doctor considers necessary to mention. To inform public authorities, medical staffs, fisheries services and private individuals about the evolution of CFP risk in French Polynesia, and improve public awareness about potential new poisoning risks, an annual report based on the CFP reports collected from the five island groups of French Polynesia is published, and also made available on www.ciguatera-online.com.

In June 2014, once informed about this unusual and particularly severe mass poisoning outbreak, the Institut Louis Malardé, in collaboration with the Public Health Directorate of French Polynesia and local authorities, decided to conduct extensive investigations in order to elucidate *T. niloticus* implication in this event. Additional expertise from Pavia Poison Control Centre, National Toxicology Information Centre (PPCC, Italy) was sought after one of the patients was transferred to their unit for medical investigation and advanced surveillance.

### 5.2. Acute Phase Description

The clinical description of acute manifestations of the poisoning (<2 months) was based on the data obtained from the Louis Rollin Hospital (Nuku Hiva, Marquesas Archipelago, French Polynesia) and the Hospital Center of French Polynesia (Tahiti, Society Archipelago) medical files, CFP-specific reporting forms, and information directly collected from patients on a questionnaire basis.

### 5.3. Six and 20-Month Medical Follow-Up

A targeted follow-up was performed at six and twenty months post-poisoning for all cases involved in the poisoning outbreak. This follow-up relied on a specific questionnaire developed by the research team from Institut Louis Malardé (ILM, Tahiti, Society Archipelago, French Polynesia) and the medical experts from Pavia Poison Control Centre, the National Toxicology Information Centre (PPCC, Italy), and approved by the Ethic Comity Salvatore Maugeri Fondazione, Italy (ethical approval number: 1047 CE). Extensive information about the age, sex, medical background before the poisoning event occurred, the context of poisoning, acute and chronic symptoms (nature, intensity, and duration), triggering factors, treatments tested in acute and chronic phases, and their respective efficiency were recorded. Patients were also asked to provide any additional information they considered useful to mention. Questionnaires were transmitted by email, except for patients no. 1 and no. 2, who were interviewed face-to-face in Tahiti for the six-month follow-up.

### 5.4. Index Case Description (Patient No. 9)

This section provides a description of the medical “journey” of patient no. 9 after being taken into the immediate care of medical professionals during the post-acute and chronic phases of the poisoning. This case description is based on data compiled from medical records containing the clinical picture at admission and during hospital stay, treatments, clinical follow-up, and the outcome from the Louis Rollin Hospital (Nuku Hiva, Marquesas Archipelago, French Polynesia), the Hospital Center of French Polynesia (Tahiti, Society Archipelago, French Polynesia) and the peripheral hospital in Northeast Italy where he was successively hospitalized before his transfer to the PPCC for medical surveillance and extensive investigation. The PPCC is a hospital-based unit (24/7) where medical clinical toxicologists advise on and assist, for diagnosis and management purposes, all physicians requiring specialist consultation from emergency departments and intensive care units all over Italy. Medical management, neurological exploration, and the evolution of the patient’s health condition at the PPCC for the six first months post-poisoning, were described for patient no. 9.

### 5.5. CTX Extraction and Detection from Tectus niloticus Samples

Toxicological investigations where conducted on 70 specimens of *T. niloticus* collected in Anaho Bay, 1, 6 and 28 months after the poisoning outbreak. Briefly, the whole animal was first extracted following the protocol of Roué et al., 2016 (i.e., twice in methanol (MeOH) and twice in 50% aqueous MeOH) [42], then, *T. niloticus* extracts were analyzed for their toxicity using the neuroblastoma cell-based assay (CBA-N2a). Finally, CTX identification was assessed by liquid chromatography coupled to tandem mass spectrometry (LC-MS/MS). The methods used are described in detail in Darius et al., 2018 [51].

## Figures and Tables

**Table 1:** Clinical manifestations expressed during the acute phase.

Patient	1	2	3	4	5	6	7	8	9
Sex	F	M	M	M	M	F	F	M	M
Age	59	65	72	50	53	47	39	58	45
Health condition before poisoning	E	G	G	E	E	E	E	E	E
Notable medical history	no	RC	HT	no	no	HT	no	no	no
Symptoms onset (hours)	2	2	2	2	2	2	4	4	2
Gastrointestinal manifestations									
Nausea		+++	++	+		+++	+	+++	+++
Vomiting	+++		+++			+++	++	+++	+++
Diarrhea	+++	+++	+++	+++	+++	+++	+++	+++	+++
Abdominal pain	+++	++	++	+++		+++			++
Dysphagia/Hiccup			x						+++
Esophagitis									+++
Gastrointestinal symptoms duration	6 h	>1 m	48 h	2 w	12 h	48 h	18 h	18 h	>1 m
Cardiovascular manifestations									
Hypotension	+++	+++	++	+++	+++	++			++
Heart rhythm disorder	+	+++	++						
Bradycardia	+	++	++	++		++			+++
Tachycardia									
Cardiovascular symptoms duration	12 h	24 h	72 h	24 h	24 h	24 h			
Neurological manifestations									
Cold allodynia	+++	+++	+++	+++		+++	+++	+++	+++
Tingling of extremities	+++	+++	x	+++		+++	+++	+++	+++
Touch disturbances	+++	+++		x		+++	+	+++	+++
Itching	+++	+++	+++			+++	+++	++	++
Dysgeusia	x	x	x	x		+		++	+
Burning sensation (throat, mouth)	x	x	x	x		+	+++	++	+
Dizziness			x			++	+		
Vision disorder	x	x	x	x		+			++
Language disorder			x			+		++	
Headache			x			++			
Balance disorder			x			++			
Anxiety							x	x	
Others									
Asthenia	+++	+++	+++	+++		+++	+++	+++	+++
Myalgia	+++	+++	x	x		++	+++	++	++
Urogenital disturbances, burning, pain	x	x	x	++		++		++	+++
Arthralgia	+	+++	++	x		++			++
Sleep disorder							+++	+++	
Hypothermia			++	++		+++			+++
Shivers						+++			+++
Feet swelling, burning, pain	x	x				x	x	x	

F: female. M: male. E: excellent. G: good. HT: hypertension. RC: renal colic. Severity classification: +: mild; ++: moderate; +++: severe; x: symptom expressed without severity information. h: hour; d: day; w: week; m: month. Underlining: Neurological or other manifestations already expressed 24 h after the poisoning, according to the CFP reporting forms filled in by medical professionals of Louis Rollin Hospital of Nuku Hiva.

**Table 2:** Clinical manifestations reported during the 6- and 20-month follow-up.

Patient	1		2		3		4		5		6		7		8		9	
Follow-Up (Month)	6	20	6	20	6	20	6	20	6	20	6	20	6	20	6	20	6	20
Gastrointestinal manifestations																		
Diarrhea			+++															
Neurological manifestations																		
Tingling of extremities	+++		+++						+++		+++			++	+	+	+++	
Touch disturbance	+++		+++						+		+++				++	+	++	
Cold allodynia	+++		+++		++		+	+			+++		++		++		++	
Itching	+++		+++		++				+++		+++		++	+	++			
Balance disorder															+			
Dysgeusia															+			
Burning (throat, mouth)													+++	+	++	+	++	
Urogenital disturbances, burning, pain																	+++	
Others																		
Asthenia	+				+				+++		++				+		++	
Myalgia											+				+++		++	
Sleep disorder	+++								+		++		+				+++	
Irritability			+++								+						+	
Shivers											++						++	
Hypothermia								+			++						++	
Feet swelling, burning, pain											x		+++	+				
Restless legs syndrome														+				

Severity classification +: mild; ++: moderate; +++: severe; x: symptom expressed without severity information.

**Table 3:** Factors responsible for the recurrence and/or intensification of clinical manifestations of the poisoning, based on the 6-months medical follow-up.

Factors	Patient No.								
	1	2	3	4	5	6	7	8	9
Alcohol	+	+			+	+	+	+	
Fish					+	+			
Beef	+	+	+	+	+	+	+	+	
Pork							+	+	
Nuts	+	+							
Food rich in protein					+	+			
Cheese			+						
Salted biscuits	+								
Physical activity							+	+	
Ambient temperature variation						+	+	+	
Fatigue, lack of sleep							+	+	
Rapid weight loss	+	+			+	+			
Wind, sun exposure							+		

+: factor responsible for the recurrence and/or intensification of clinical manifestations according to patients
